# Inhibition of mitochondrial carrier homolog 2 (MTCH2) suppresses tumor invasion and enhances sensitivity to temozolomide in malignant glioma

**DOI:** 10.1186/s10020-020-00261-4

**Published:** 2021-01-28

**Authors:** Qiuyun Yuan, Wanchun Yang, Shuxin Zhang, Tengfei Li, Mingrong Zuo, Xingwang Zhou, Junhong Li, Mao Li, Xiaoqiang Xia, Mina Chen, Yanhui Liu

**Affiliations:** Department of Neurosurgery, State Key Laboratory of Biotherapy, West China Hospital, Sichuan University, Chengdu, 610041 People’s Republic of China

**Keywords:** MTCH2, Glioma, Temozolomide, Mitochondria, Cell migration/invasion, Cell death

## Abstract

**Background:**

Malignant glioma exerts a metabolic shift from oxidative phosphorylation (OXPHOs) to aerobic glycolysis, with suppressed mitochondrial functions. This phenomenon offers a proliferation advantage to tumor cells and decrease mitochondria-dependent cell death. However, the underlying mechanism for mitochondrial dysfunction in glioma is not well elucidated. MTCH2 is a mitochondrial outer membrane protein that regulates mitochondrial metabolism and related cell death. This study aims to clarify the role of MTCH2 in glioma.

**Methods:**

Bioinformatic analysis from TCGA and CGGA databases were used to investigate the association of MTCH2 with glioma malignancy and clinical significance. The expression of MTCH2 was verified from clinical specimens using real-time PCR and western blots in our cohorts. siRNA-mediated MTCH2 knockdown were used to assess the biological functions of MTCH2 in glioma progression, including cell invasion and temozolomide-induced cell death. Biochemical investigations of mitochondrial and cellular signaling alternations were performed to detect the mechanism by which MTCH2 regulates glioma malignancy.

**Results:**

Bioinformatic data from public database and our cohort showed that MTCH2 expression was closely associated with glioma malignancy and poor patient survival. Silencing of MTCH2 expression impaired cell migration/invasion and enhanced temozolomide sensitivity of human glioma cells. Mechanistically, MTCH2 knockdown may increase mitochondrial OXPHOs and thus oxidative damage, decreased migration/invasion pathways, and repressed pro-survival AKT signaling.

**Conclusion:**

Our work establishes the relationship between MTCH2 expression and glioma malignancy, and provides a potential target for future interventions.

## Background

Originating from glial cells, glioma is a common type of malignant and lethal tumor in the brain, which can be divided into astrocytomas, oligodendrogliomas, and glioblastomas (GBM) (Omuro and DeAngelis 2013; Laug et al. 2018). Glioma displays aggressive growth and diffuse invasion, and evolves rapidly by acquiring new mutations and oncogene expressions for drug resistance (Puchalski et al. 2018). Although current standard care of maximal safe surgical resection followed by radiotherapy and concurrent temozolomide (TMZ) chemotherapy provides survival benefit to selected patients (Stupp et al. 2009), glioma still exhibits poor responses to approved therapies and carries an inferior prognosis. Therefore, molecular understandings of the initiation and progression of glioma are urgently needed.

Over the last decades, a well-appreciated hallmark of glioma is the altered tumor metabolism (Agnihotri and Zadeh 2016; Venneti and Thompson 2017), a phenomenon known as Warburg effect (Warburg et al. 1927). This effect fulfills the demands for energy and biomass production of rapid proliferation in glioma cells (Venneti and Thompson 2017). Extensive studies established that mitochondrial dysfunction is a prominent mechanism by which glioma cells exert its metabolic shift from oxidative phosphorylation (OXPHOs) to glycolysis, regardless of oxygen availability. During this metabolic reprogramming, mitochondrial function in glioma is evidently suppressed through mutations of mitochondrial DNA (Keatley et al. 2019), altered metabolic enzyme atlas (Deighton et al. 2014; Franceschi et al. 2018), and imbalanced morphological dynamics (Xie et al. 2015). All these events offer a proliferation advantage to tumor cells and decrease mitochondria-dependent cell death to chemical drugs (Vyas et al. 2016). Although a number of studies have increased the understanding of mitochondrial dysfunction in glioma cells, the molecular repertoire is still largely unknown.

To explore the mechanistic interplay between mitochondrial dysfunction and glioma progression, we focused on mitochondrial carrier homolog 2 (MTCH2), a critical regulator of mitochondrial metabolism and related cell death (Robinson et al. 2012; Veresov and Davidovskii 2014). MTCH2 is a mitochondrial outer membrane protein that interacts with the truncated BH3-interacting domain death agonist (tBID) to regulate cell apoptosis (Cogliati and Scorrano 2010; Katz et al. 2012; Zaltsman et al. 2010). Previous studies demonstrate that loss of MTCH2 impairs mitochondrial architecture and functions, including enlarged size (Bahat et al. 2018), reduced motility (Ruggiero et al. 2017), and elevated oxidative stress (Buzaglo-Azriel et al. 2017). MTCH2 expression was associated with several types of tumors. For example, MTCH2 was down-regulated in ErbB2-driven mammary carcinoma (Arigoni et al. 2013), and its induction reduced tumorigenicity and leads to growth arrest of breast cancer (Leibowitz-Amit et al. 2006). A recent study showed the function of MTCH2 in acute myeloid leukemia (AML). Inhibiting MTCH2 decreased tumor growth and induced differentiation of AML cells (Khan et al. 2020). However, the role of MTCH2 in malignant glioma is not defined.

In this study, we reveal the novel function of MTCH2 in malignant glioma. MTCH2 expression is closely associated with glioma progression, and its knockdown impairs cell migration and enhances temozolomide sensitivity of glioma cells. Our work establishes the relationship between MTCH2 expression and glioma malignancy, and defines MTCH2 as a potential target for future interventions.

## Methods

### Patient samples

Glioma and adjacent brain tissues included in this study were obtained from primary glioma patients undergoing elective craniotomy at West China Hospital between January 2019 and January 2020 (Additional file 1: Table S1). All human studies were approved by the Institutional Review Board of West China Hospital of Sichuan University (project identification code: 2018.569). All patients provided written informed consent. The diagnoses of glioma grade were made by three independent pathologists. We used fluorescence-guided surgery (FGS), e.g. 5-aminolevulinic acid (5-ALA) to achieve intraoperative visualization of tumor tissue under the microscope and distinguish the tumors from normal adjacent tissues. Clinical samples obtained from patients were used for detection the mRNA and protein levels.

### Bioinformatic analysis

Biomarker data and clinical information of The Cancer Genome Atlas Research Network (TCGA) and the Chinese Glioma Genome Atlas (CGGA) LGG-GBM cohorts were downloaded from TCGA (https://portal.gdc.cancer.gov) and CGGA (http://www.cgga.org.cn) repository. Normalized RNA-seq data of 708 samples from 681 cases in TCGA were downloaded using R with the TCGAbiolinks package. And mRNAseq_325 data of 325 samples from 325 cases in CGGA were also downloaded. IDH mutation was determined by merging IDH1 codon 132 and IDH2 codon 172 mutations. Chromosome 1p19q co-deletion was determined by 1p32 and 19q13 segment focal CNV value less than -0.3. MGMT promoter methylation level bins were divided using the 10% and 40% percentiles of mean beta value of probes between 131264896 and 131265737 on chromosome 10 (GRCh38 genome). For survival analysis, patients were divided into low and high gene expression groups by median expression level or 95% percentile of MTCH2 expression in normal brain tissue. Hazard ratio for expression was tested using log rank test. Gene expression is presented as normalized FPKM. Correlation and survival analysis were conducted in R using grid and survminer packages.

### Cell culture and siRNAs transfection

Human glioblastoma cell lines U-87 MG, U-251 MG and A172 cell lines were obtained from Type Culture Collection of the Chinese Academy of Sciences (CTCC, Shanghai, China). Glioma cells were maintained at DMEM (Hyclone) with 10% FBS (PAN), and cultured under 5% CO_2_ at 37 °C incubator. All cells were validated by Short Tandem Repeat (STR) analysis. For MTCH2 knockdown, targeted siRNA were transfected into cultured glioma cells using Lipofectamine RNAiMAX reagent (Invitrogen) in Opti-MEM (Invitrogen) over 72 h. The siRNA sequences are as follow: siRNA MTCH2#1: sense 5′-GGUAAAGUUUUACAGCAUUTT-3′; antisense 5′-AAUGCUGUAAAACUUUACCAT-3′; siRNA MTCH2#2: sense 5′-GAGCCGAGGAAAUAGCUUATT-3′; antisense 5′- UAAGCUAUUUCCUCGGCUCAT-3'.

### Primary cell culture of mouse neurons and astrocytes

To compare the MTCH2 protein levels in primary brain cells with glioma cells, we set up experiments to culture primary mouse neurons and astrocytes. Mouse cortical neuron cultures were performed following the standard methods (Kaech and Banker 2006). Briefly, cerebral cortex tissues from mouse embryos of 18.5 days were dissected and digested with Papain and DNAse. Then digested neurons were plated in 6-well culture plates with a density of 1 × 10^6^ cells per well and harvested at DIV 14 for Western blots (Tan et al. 2019). Primary mouse astrocytes were cultured from cerebral cortex from newborn mouse as described, and digested cells were planted in MEM supplemented with 10% Horse Serum and antibiotics. Cultured astrocytes were then harvested for Western blots.

### Immunofluorescence

For immunofluorescence assay to observe MTCH2 cellular localization, A172 cells were cultured on coverslips over 48 h. For Mito-Tracker staining, A172 cells were incubated with the Mito-Tracker dye (Invitrogen, 200 nM) in DMEM at 37 °C incubator for 15 min (mins). Then, cells were fixed with 4% paraformaldehyde (PFA, Sigma) solution at room temperature for 30 min. After permeabilization with 0.25% Triton X-100 in PBS, cells were blocked with 10% goat serum and incubated with primary antibodies (anti-MTCH2, Proteintech) overnight at 4 °C. For F-actin staining, 20 nM rhodamine-phalloidin (Thermo) diluted with PBS was applied to PFA-fixed cells that were permeabilized. Then cells were washed with PBS for three times and incubated with ALEXA FLUOR 488/594 secondary antibodies, and mounted slides using ProLong® Gold Antifade Reagent with DAPI (Invitrogen). Images were acquired using Olympus BX63 microscope.

### Cell apoptosis assay

To detect cell apoptosis, we conducted flow cytometry and Hoechst staining in glioma cells treated with temozolomide (Selleck Chemicals, 200 μM). For flow cytometry, glioma cells were cultured with 60 ~ 80% density in 6-well plate. After cells were transfected with siRNA-MTCH2 and subsequent temozolomide treatment for 48 h, cells were detached by 0.25% trypsin (Invitrogen) and applied with Annexin V Alexa Fluor647/7-AAD kit (Beijing 4A Biotech) for flow cytometry on a Beckman cytoflex. For Hoechst 33258 staining, cells were cultured on coverslips overnight, and treated as above. Then cells were fixed by 4% paraformaldehyde (PFA) solution and incubated with Hoechst 33258 staining kit (Beyotime) as manufacture instructions. Images were acquired using Olympus BX63 microscope, and cell counting was performed by Image Pro Plus software.

### Scratch wound healing assay

To detect the effect of MTCH2 on cell migration of glioma cells, we performed scratch wound healing assay as previous described (Li et al. 2020). Briefly, glioma cells were seeded in 6-wells plate overnight, and then siRNA MTCH2 were transfected into cells when cells reached at least 90% density. Cells were wounded with a 200 μL pipette tip and washed 3 times by PBS to remove detached cells. During the migration, glioma cells were cultured in DMEM without FBS. Images were continuously captured in period days and analyzed by ImageJ software.

### Transwell migration and invasion assay

To evaluate the effect of MTCH2 on cell migration/invasion of glioma cells, we performed transwell migration and invasion assays, using chambers (8.0 μm) in 24-well plates (Corning, USA). For migration assay, U87 MG cells were seeded in the upper chamber with a density of 2 × 10^4^ cells/well, and the lower filled with DMEM containing 5% FBS as a chemoattractant. After 8 h, the upper surface of the membrane was scrubbed to remove all non-invaded cells. Cells migrated to the lower surface were fixed and stained with 0.5% crystal violet solution. For invasion assay, cells were seeded into the upper chamber pre-coated with BioCoat Matrigel (BD, USA) according to the manufacturer's protocol. After 24 h, the cells invaded were fixed and stained with crystal violet. The invaded cells were calculated by ImageJ and quantified for statistical analysis from three independent experiments.

### Cell lysis solution and western blots

Total cells were lysed with 2% SDS solution plus protease and phosphatase inhibitors (Thermo Scientific). Protein concentrations were tested by BCA kit and equivalent proteins were loaded into SDS-PAGE. Following western blots were performed according to standard procedures. The primary antibodies were list as follow: MTCH2 (Proteintech, Cat#16888-1-AP), Beta actin (Boster Biological Technology, Cat#BM0627), PDHE1-A (Abcam, Cat#ab110334), GFAP (Millipore, Cat#G3893), Tom20 (Santa Cruz Biotechnology, Cat#sc-136211), Tom40 (Abcam, Cat#ab185543), MMP-9 (Cell Signaling Technology, Cat#3852), N-cadherin (Cell Signaling Technology, Cat#4061), Vimentin (Cell Signaling Technology, Cat#5741), 4-HNE (Abcam, Cat#ab48506), BcL-2 (Santa Cruz Biotechnology, Cat#sc-7382), Bax (Millipore, Cat#06-499), phospho-AKT (Thr308) (Cell Signaling Technology, Cat#2965), phospho-AKT (Ser473) (Cell Signaling Technology, Cat#4060), AKT (Cell Signaling Technology, Cat#9272), phospho-S6 (Ser240/244) (Cell Signaling Technology, Cat#2215), S6 (Cell Signaling Technology, Cat#2217), phospho-4EBP1 (Thr37/46) (Cell Signaling Technology, Cat#9459), total OXPHOs rodent antibody cocktail (Abcam, Cat#110413), phospho-Cofilin1 (Ser3) (Cell Signaling Technology, Cat#3313), Cofilin1 (Cell Signaling Technology, Cat#5175), PARP (Abclonal, Cat#A0942) and cleaved caspase 3 (Cell Signaling Technology, Cat#9661).

### RNA extraction and real-time PCR

Trizol reagent was used to extract total RNA following manufacturer instructions. RNA reversed transcription using PrimeScriptTM RT reagent Kit (Takara), and analyzed by quantitative PCR (qPCR) using SYBR Premix Ex TaqTM II (Takara) in Bio-Rad iQ5 system. Relative gene expression was normalized to β-actin. qPCR primers were as follows:

**Table:** 

Targets	Forward 5′-3'	Reverse 5′-3'
MTCH2	GGTCTTGTTCCTCGCCTTCT	TGGTAGAAACCCCACTGTCC
MMP-9	GGGACGCAGACATCGTCATC	TCGTCATCGTCGAAATGGGC
β-actin	CATGTACGTTGCTATCCAGGC	CTCCTTAATGTCACGCACGAT

### Statistical analysis

Statistical analysis was conducted in GraphPad Prism (v6.01, GraphPad Software, Inc). Data were expressed as mean ± SEM. Differences between groups were tested for statistical significance using Student’s *t* test for two-group comparisons, and one-way analysis of variance (ANOVA) followed by the Tukey post hoc tests for multi-group comparisons. A p-value < 0.05 (*) was considered statistically significant, and **indicates p < 0.01, and ***p < 0.001.

## Results

### MTCH2 expression is associated with glioma malignancy.

**Fig. 1:**
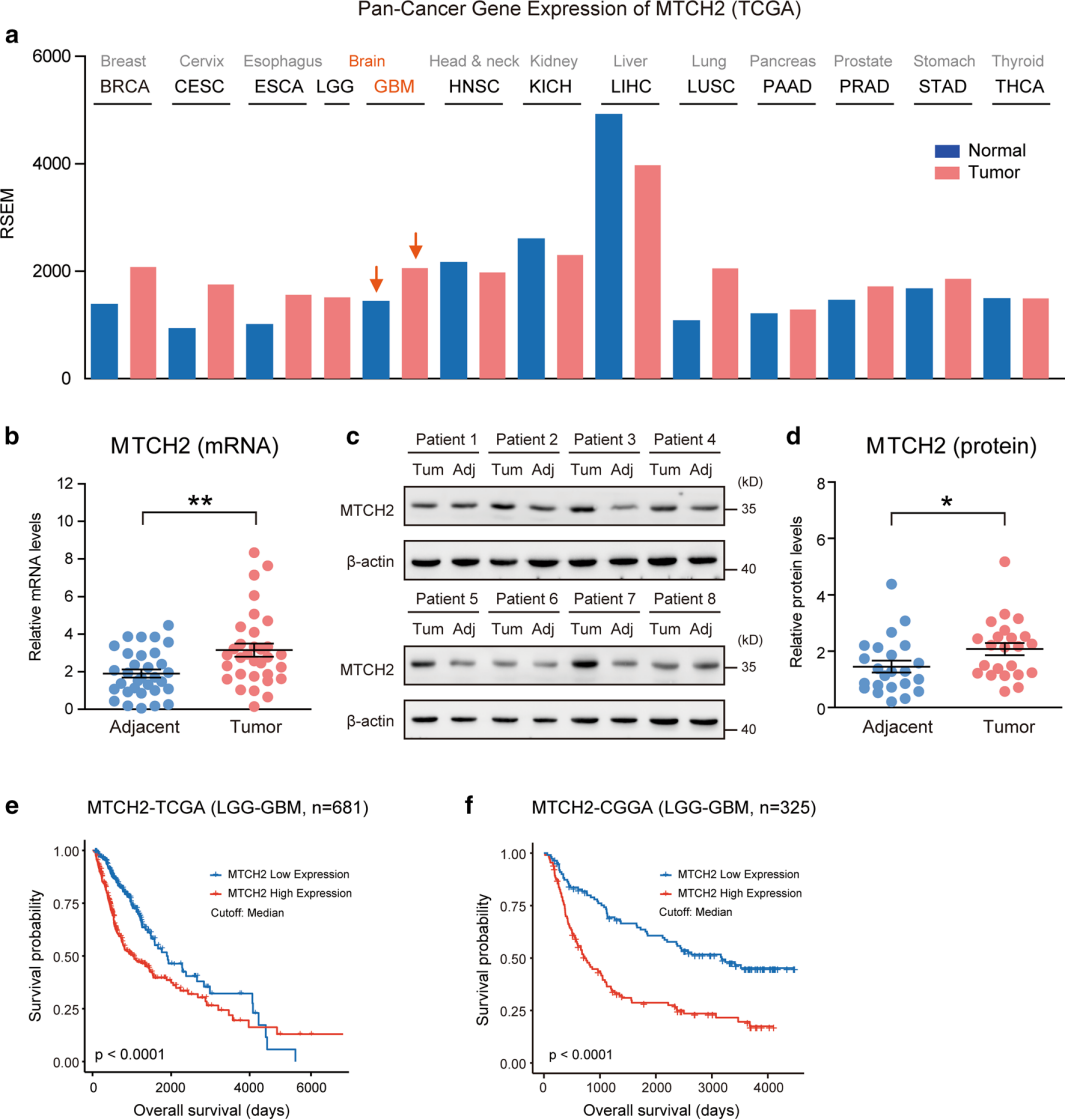
MTCH2 expression is increased in glioma tissues and indicates poor prognosis. **a** A Pan-cancer diagram showing the increased MTCH2 expression in brain gliomas. **b**Real-time PCR results showing increased mRNA level of MTCH2 in glioma tissues compared to paired adjacent brain tissues (n = 32). **c**, **d** Western blots (**c**) and quantifications (**d**) showing increased proteins of MTCH2 in glioma tissues compared to paired adjacent brain tissues (n = 23). **e**, **f** Survival analysis using clinical information from TCGA (**e**) and CGGA (**f**) dataset. Patients are divided into low and high MTCH2 groups by median expression level

As a first step to investigate the possible contribution of MTCH2 in gliomagenesis, we analyzed MTCH2 expression profiles across multiple types of human tumors. Interrogation of TCGA Pan-Cancer dataset revealed that MTCH2 was highly expressed in several tumors despite different tissue origins, including cancers in the breast (BRCA), cervix (CESC), esophagus (ESCA), lung (LUSC) and brain (GBM) (Fig. 1a). This data suggests that MTCH2 overexpression may be a general mechanism employed by solid tumors. Further examinations confirmed that the mRNA and protein levels of MTCH2 were increased in the tumor compared to matched adjacent tissues in our glioma cohort (Fig. 1b–d; Additional file 1: Table S2). Consistent with the fact that MTCH2 expression increased in glioma tissues, Kaplan–Meier survival analysis for multiple human glioma datasets (TCGA and CGGA) showed that patients with lower MTCH2 expression displayed significantly better overall survival than those with higher MTCH2 expression (Fig. 1e–f). Therefore, we conclude that MTCH2 is highly expressed in human gliomas and its expression reciprocally correlates with patient survival (Additional file 2: Fig. S1 and Additional file 1: Table S2).

**Fig. 2:**
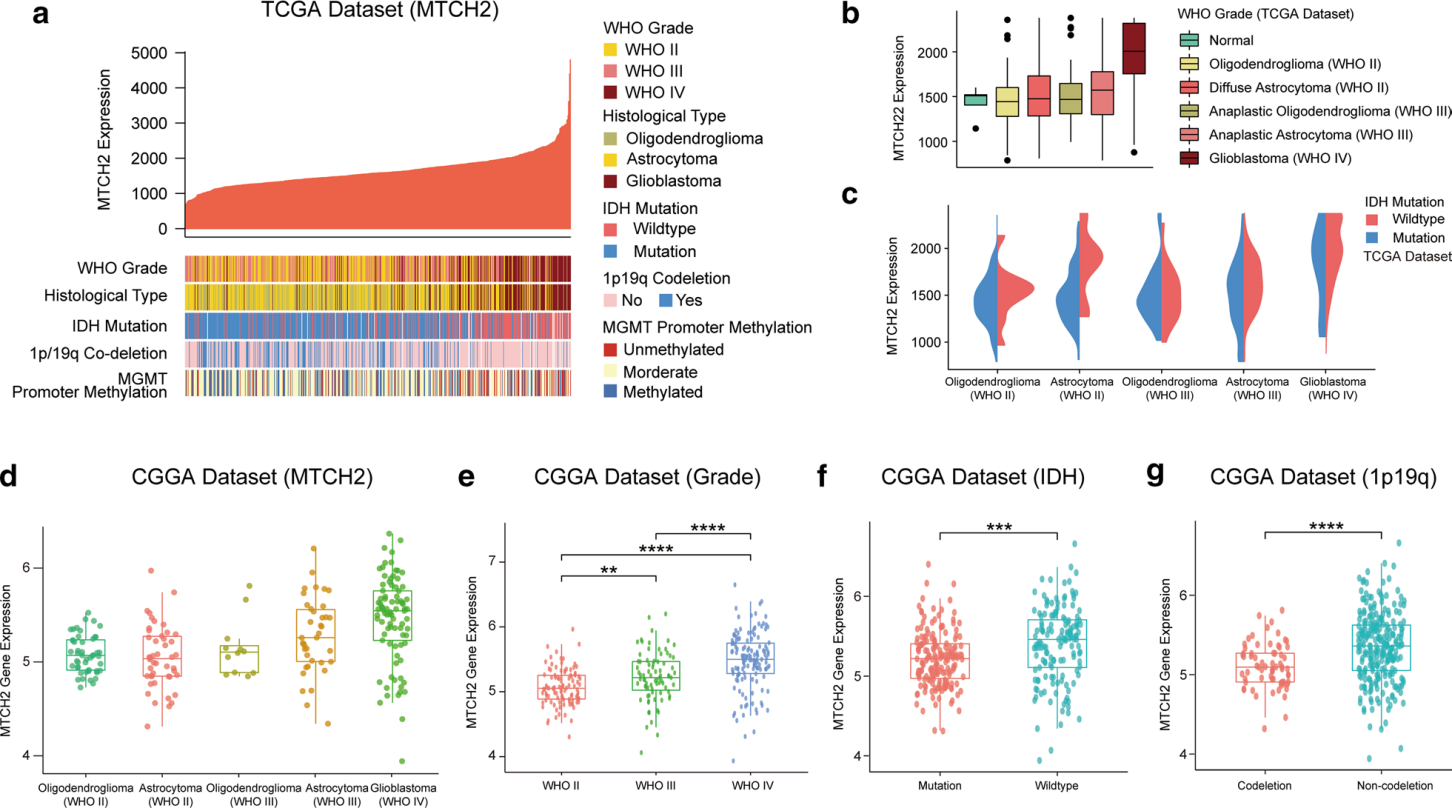
MTCH2 expression is associated with glioma malignancy. **a** Correlation between MTCH2 expression (FPKM) and glioma histology and prognostic biomarkers in The Cancer Genome Atlas (TCGA) dataset. Patients (N = 681) are sort by MTCH2 expression. Glioma histology and biomarkers of patients are shown in color code. **b** Diagrams showing MTCH2 expressions in glioma histology groups. **c** Diagrams showing MTCH2 expression of gliomas with IDH status in the TCGA dataset. **d**–**g** Diagrams showing MTCH2 expression of gliomas with tumor grade, histology, IDH and 1p19q status in the CGGA dataset

To further investigate the correlation of MTCH2 expression with clinical features in glioma, we performed an in silico analysis using gene expression and associated clinical data of glioma cohorts from two independent publicly available datasets, TCGA and CGGA. Results from TCGA showed that MTCH2 expression was positively correlated with glioma grade, and high MTCH2 expression tended to be associated with GBM subtype (Fig. 2a, b), coinciding with the fact that MTCH2 correlates with poor patient survival. For human glioma, IDH mutation, chromosome 1p/19q co-deletion and MGMT promoter methylation are pivotal biomarkers for the guidance of glioma prognostication and treatment (Eckel-Passow et al. 2015). We found that MTCH2 expression was exceedingly associated with IDH wildtype and 1p/19q non-codeletion group, which indicates poor glioma prognosis (Fig. 2a, c). Consistently, data from CGGA confirmed that MTCH2 expression was positively associated with tumor grade, IDH wildtype and 1p/19q non-codeletion in Chinese glioma population (Fig. 2d–g), supporting the notion that MTCH2 expression is correlated with glioma malignancy.

### Silencing MTCH2 expression may increase mitochondrial OXPHOs in human glioma cells.

**Fig. 3:**
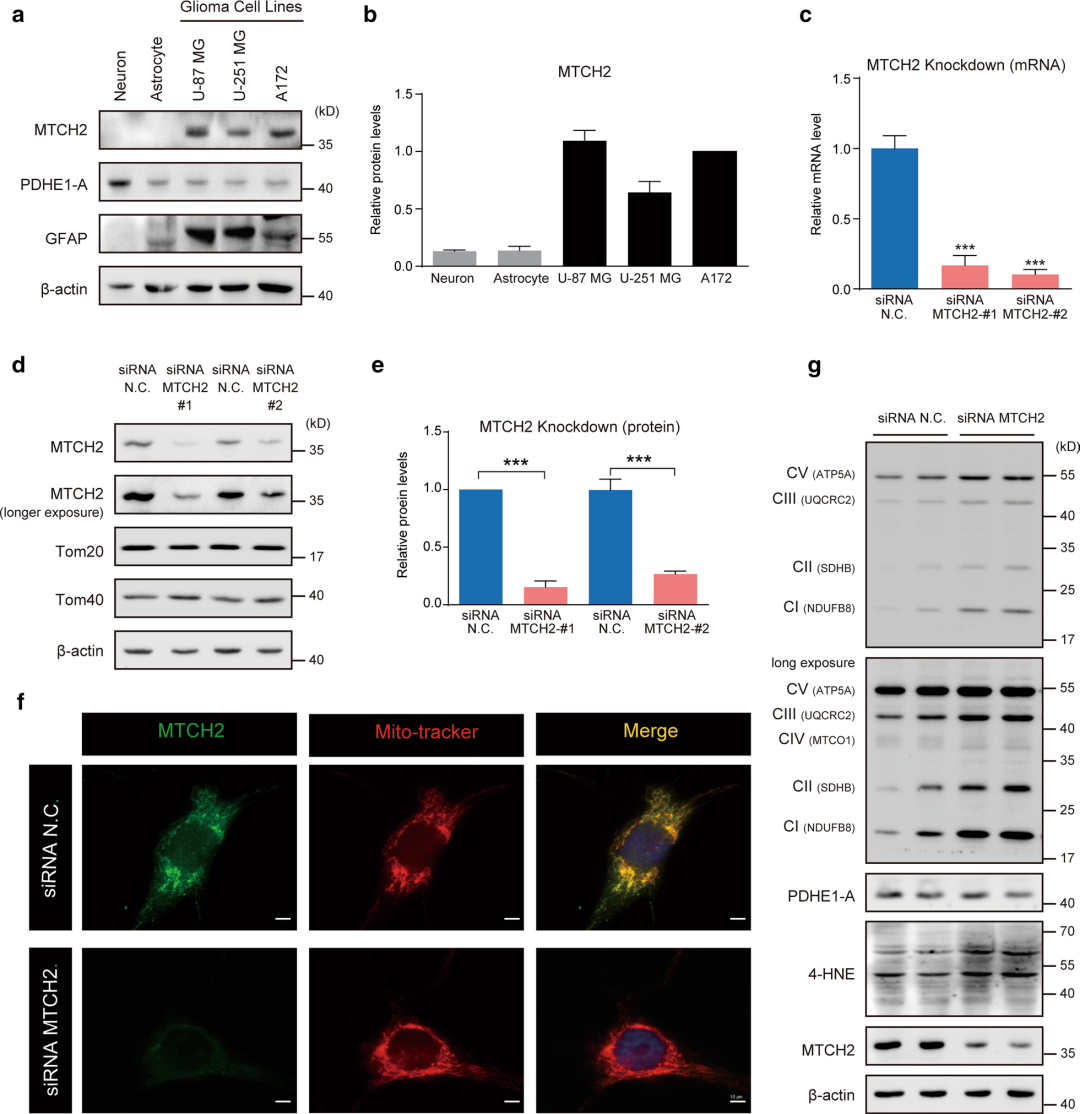
High expression of MTCH2 in glioma cells may increase mitochondrial OXPHOs. **a**, **b** Western blots and quantifications showing the enriched expression of MTCH2 in glioma cells compared to primary neurons and astrocytes. **c** qPCR results showing the decreased mRNA level of MTCH2 in A172 cells by siRNA-mediated knockdown (N = 3). **d**, **e** Western blots and quantifications showing the decreased protein level of MTCH2 in A172 cells by siRNA-mediated knockdown. Noted that other mitochondrial outer membrane proteins Tom20 and Tom40 were not affected by MTCH2 knockdown. **f** Immunostaining images showing the mitochondrial localization of MTCH2 in A172 cells. Noted that MTCH2 signals were dramatically decreased by siRNA-mediated knockdown. Scale bar, 10 μm. **g** Western blots showing the increased mitochondrial respiratory proteins and 4-HNE levels by MTCH2 knockdown in A172 cells

To explore the biological function of MTCH2 in glioma cells, we first examined its expression pattern in glioma cell lines, compared with primary cultured brain cells, including neurons and astrocytes from embryonic mouse. Western blot results showed that MTCH2 protein was extremely high in glioma cell lines, including U-87 MG, U-251 MG and A172 cells, in contrast to primary mouse neurons and astrocytes (Fig. 3a, b). Noted that all the three glioma cells were GFAP positive, whereas neurons were enriched with PDHE1-A, a metabolic gatekeeper in mitochondrial oxidative phosphorylation **(**Additional file 2: Fig. S2a-b) (Michelakis et al. 2010). We next conducted a knockdown strategy using small interfering RNA (siRNA) to reduce MTCH2 expression in A172 human glioblastoma cells. Real-time PCR results showed that the mRNA level of MTCH2 was dramatically decreased by targeted siRNA (Fig. 3c). Western blot results further confirmed that MTCH2 proteins were selectively decreased by > 70% in siRNA-MTCH2 groups (Fig. 3d–e; Additional file 2: Fig. S2c–d). Immunofluorescence images revealed that MTCH2 signals were well co-localized with Mito-Tracker, and these signals were dramatically reduced by MTCH2 knockdown (Fig. 3f).

Previous studies have extensively showed that MTCH2 knockdown/knockout increased mitochondrial OXPHOs (Buzaglo-Azriel et al. 2017; Maryanovich et al. 2015). To further address the question that whether MTCH2 knockdown increases mitochondrial respiratory function in glioma cells, here, we compared expression profiles of the respiratory chain complexes using the OXPHOs antibody cocktail (ab110413, Abcam) by Western blot. Data showed that MTCH2 knockdown increased the protein levels of OXPHOs, including NDUFB8 (complex I, CI), SDHB (complex II, CII), UQCRC2 (complex III, CIII), MTCO1 (complex IV, CIV), and ATP5A (complex V, CV) (Fig. 3g; Additional file 2: Fig. S2e). These data suggest that MTCH2 knockdown may increase OXPHOs in glioma cells.

As a result of increased mitochondrial respiratory, MTCH2 knockdown generally boosts 4-Hydroxy-2-Nonenal (4-HNE) levels, a marker of lipid peroxidation by reactive oxygen species (ROS) accumulation (Esterbauer et al. 1985), implying that loss of MTCH2 induces oxidative stress (Fig. 3g; Additional file 2: Fig. S2e). It's also noted that protein level of mitochondrial TCA cycle enzyme PDH was not affected by MTCH2 knockdown (Fig. 3g; Additional file 2: Fig. S2e). In summary, all these findings support the notion that MTCH2 is highly expressed in glioma cells, and may inhibit mitochondrial OXPHOs in glioma cells.

### Silencing MTCH2 expression impairs cell migration and invasion of human glioma cells.

**Fig. 4:**
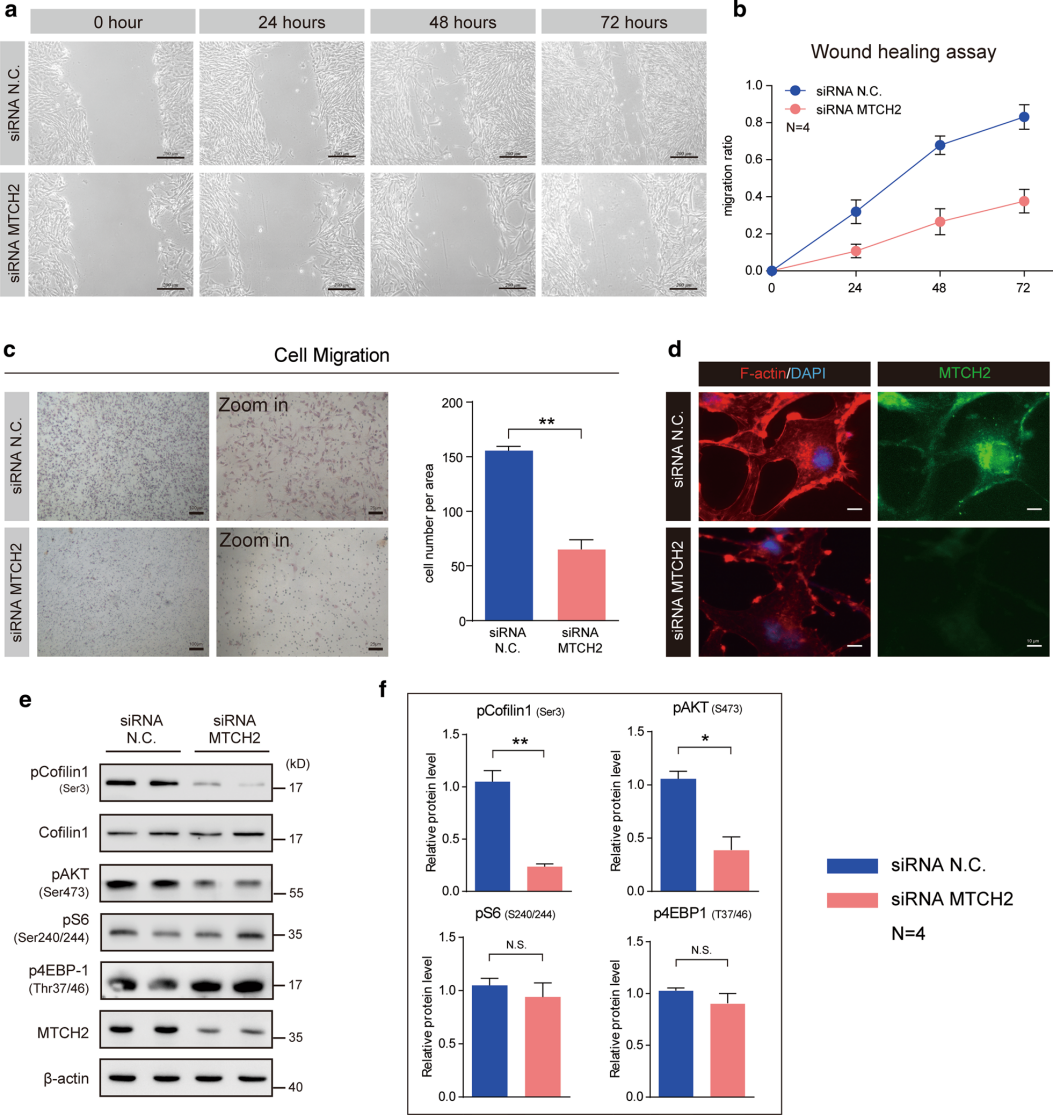
MTCH2 knockdown impairs the migration of glioma cells in vitro. **a**, **b** Representative images of wound healing assays showing that MTCH2 knockdown inhibits the migration of A172 cells (N = 4). The migration of cells into the wound was assessed at 0, 24, 48 and 72 h. Scale bar, 200 μm. **c** Images and quantifications showing that MTCH2 knockdown reduces the rate of migration of U87 MG cells by Transwell migration assays. **d** Immunostaining images showing the reduced F-actin fibers by MTCH2 knockdown in A172 cells. Scale bar, 10 μm. **e**, **f** Western blots and quantifications showing that MTCH2 knockdown alters cellular signalings relative to cytoskeleton dynamics

Based on the finding that MTCH2 expression is positively correlated with glioma malignancy, we asked whether MTCH2 functionally promotes gliomagenesis. A common feature of glioma is the diffused tumor migration/invasion, and therefore we first evaluated the effect of MTCH2 knockdown on the suppression of glioma cell migration/invasion. Images of the wound healing assay showed that MTCH2 knockdown dramatically reduced the migration of A172 cells (Fig. 4a, b). This effect was confirmed by the transwell migration assay in U87 MG cells, showing that the number of cells invaded through the chambers were decreased by MTCH2 knockdown, compared with wildtype U87-MG cells (Fig. 4c). Glioma migration is driven by the assembling of actin filaments (Schiapparelli et al. 2017). We found that MTCH2 knockdown decreased the fluorescence intensity of F-actin in A172 cells, suggesting that MTCH2 knockdown impairs the cytoskeleton dynamics in glioma cells that is required for tumor migration (Fig. 4d). Cofilin1 is one of the primary actin filament severing proteins and its activation is by dephosphorylation on serine 3 (Moriyama et al. 1996). Results showed MTCH2 decreased the phosphorylation of Cofilin1, implying increased Cofilin1 activity that depolymerizes F-actin fibers (Fig. 4e, f). Moreover, we found that mTORC2 activity (indicated by pAKT-Ser473), which is also critical in the regulation of actin dynamics (Jacinto et al. 2004), was dramatically reduced by MTCH2 knockdown (Fig. 4e, f). All these data support that silencing MTCH2 in glioma cells impairs cell migration through multiple cellular mechanisms.

**Fig. 5:**
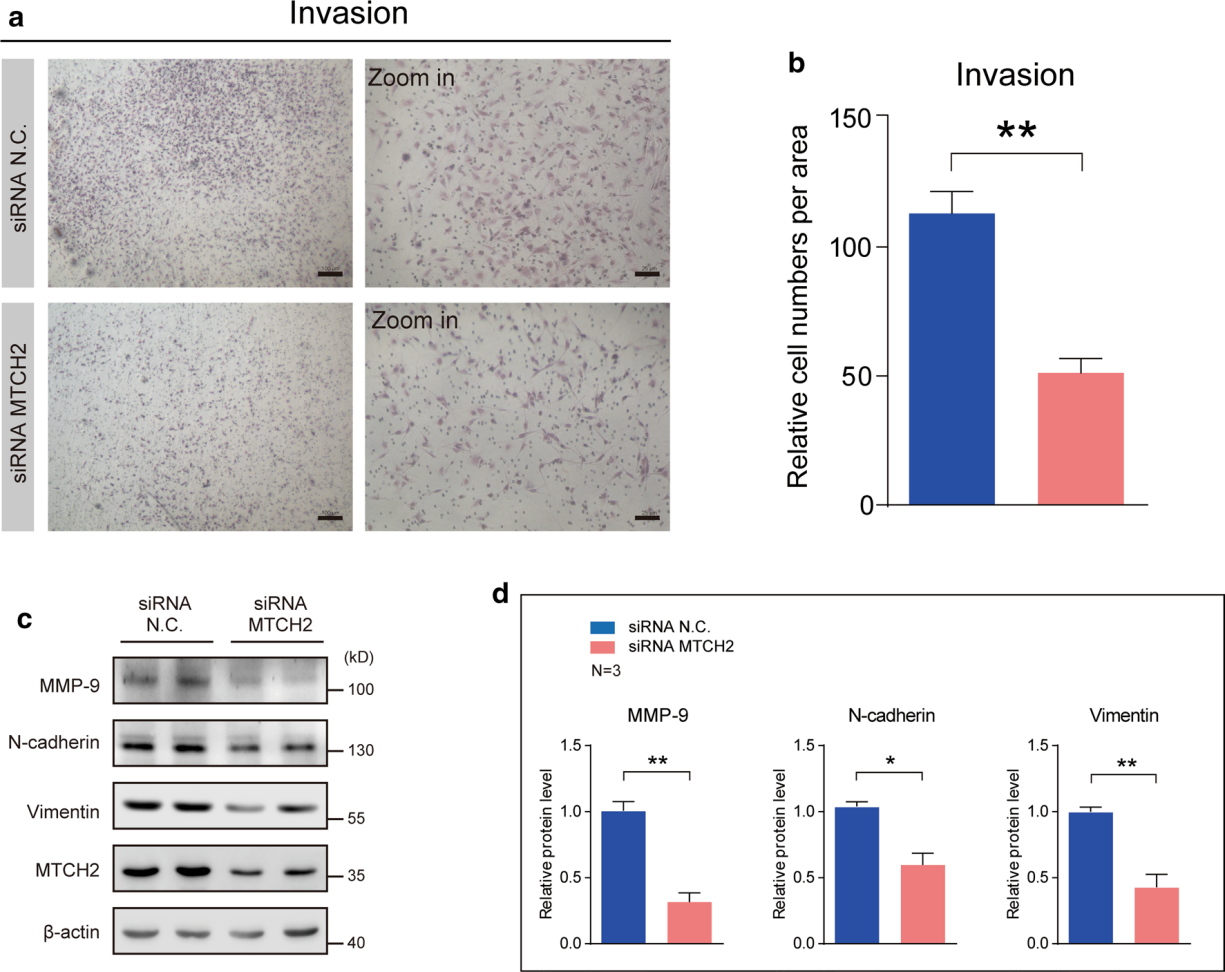
MTCH2 knockdown impairs the invasion of glioma cells in vitro. **a**, **b** Images and quantifications showing that MTCH2 knockdown reduces the rate of invasion of U87 MG cells by transwell Matrigel invasion assay. **c**, **d** Western blots (**c**) and quantifications (**d**) showing the decreased protein levels of MMP-9, N-cadherin, Vimentin and MTCH2 in A172 cells (N = 3)

To further explore the role of MTCH2 in glioma invasion, we performed transwell Matrigel invasion assay to examine whether MTCH2 knockdown inhibits the invasion of glioma cells. After 24 h of incubation, it's found that the numbers of U87-MG cells invaded through the matrigel were much less in MTCH2 knockdown groups than that in the control, suggesting that MTCH2 knockdown strongly inhibited the invasive ability of U87-MG cells (Fig. 5a, b). Biochemical results also showed that cellular signaling related to cell migration, including MMP-9, N-cadherin and Vimentin, were decreased by MTCH2 silencing (Fig. 5c, d). We further found the MMP-9 mRNA was partially reduced by MTCH2 knockdown (Additional file 2: Fig. S2e), indicating that the reduced MMP-9 expression may contribute to the decreased invasion activity of glioma cells. Taken together, all these results reveal that MTCH2 is required for the migration and invasion of glioma cells.

### Silencing MTCH2 expression enhances temozolomide sensitivity of human glioma cells

**Fig. 6:**
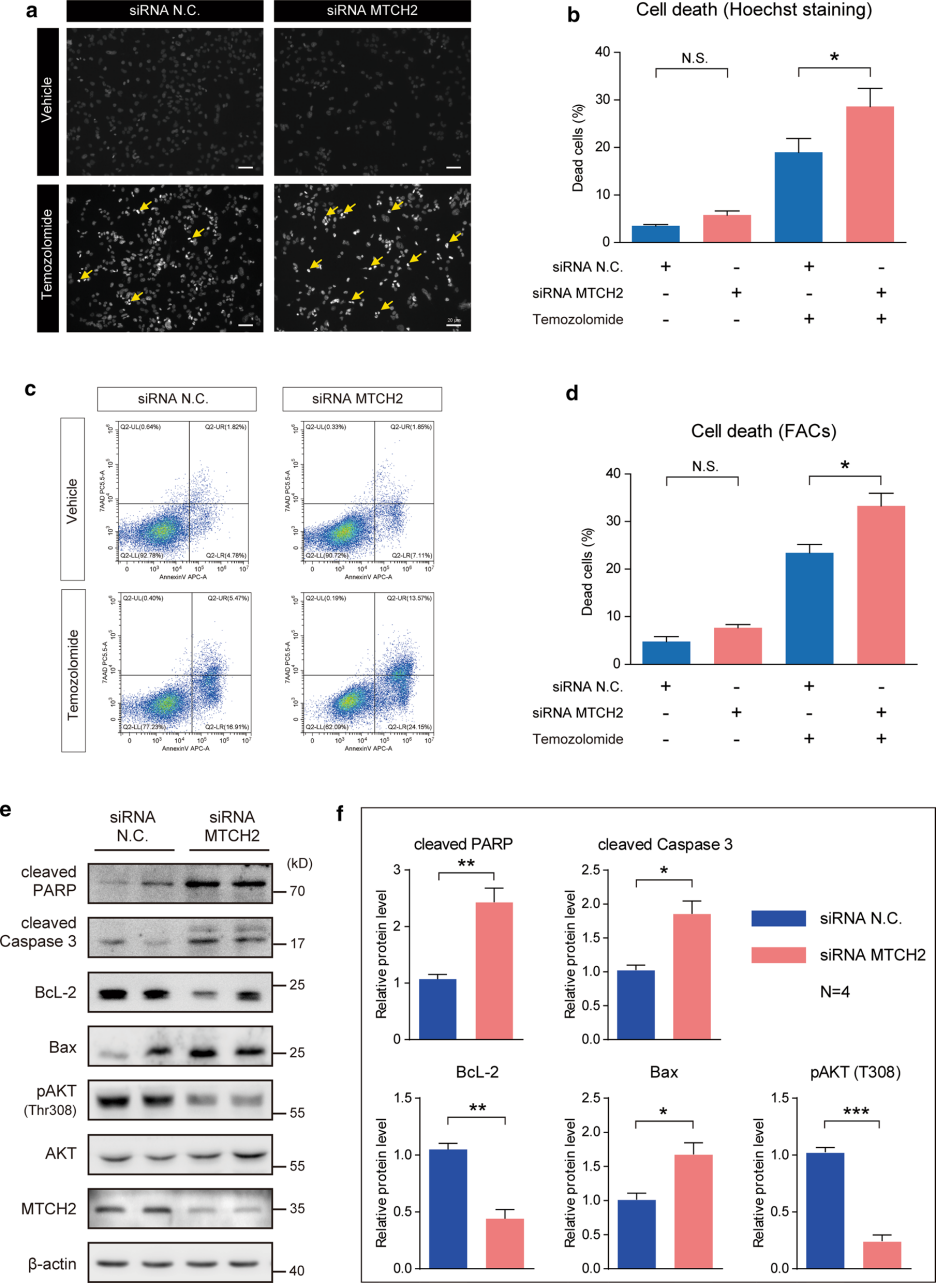
MTCH2 knockdown increases temozolomide-induced apoptosis of glioma cells.** a**, **b** Images (**a**) and quantifications (**b**) of Hoechst staining showing that MTCH2 knockdown promotes cell apoptosis of A172 cells after temozolomide treatment (200 μM, 48 h) (N = 3). Scale bar, 20 μm. **c**, **d** Images (**c**) and quantifications (**d**) of flow cytometry showing that MTCH2 knockdown promotes apoptosis of A172 cells after temozolomide treatment (200 μM, 48 h) (N = 3). **e**, **f** Western blots (**e**) and quantifications (**f**) showing that MTCH2 knockdown alters cellular signalings relative to cell survival/death (N = 4)

Another common feature of glioma is the drug resistance. The glioma resistance to temozolomide (TMZ) is the leading cause of chemotherapy failure. We investigate the effect of MTCH2 on cell survival and TMZ sensitivity in glioma cells. Under basal conditions, MTCH2 knockdown slightly affected the survival of A172 cells by Hoechst staining (Fig. 6a, b). However, when cells applied with TMZ, we found that MTCH2 knockdown dramatically increased cell death (Fig. 6a, b). Moreover, data from flow cytometry showed similar results that MTCH2 knockdown cells were more sensitive to TMZ (Fig. 6c, d). These data suggest that MTCH2 silencing enhances TMZ sensitivity in glioma cells in vitro.

MTCH2 is reported to associate with cell apoptosis involving tBID (Cogliati and Scorrano 2010; Katz et al. 2012; Zaltsman et al. 2010). Building on the observation that MTCH2 knockdown increases cell apoptosis by TMZ, we tested if MTCH2 regulates mitochondrial related cell survival/death pathways. We found that MTCH2 knockdown increases the cleavage of caspase 3 and PARP, which are executors of cell death programming (Fig. 6e, f). AKT, a serine-threonine kinase, can function to either reduce cellular destruction from oxidants and supports cell survival via BcL-2/Bax pathways (Matsuzaki et al. 1999). We found that MTCH2 silencing decreased AKT phosphorylations at Thr308, and reduced pro-survival protein BcL-2 with induced pro-apoptotic protein Bax (Fig. 6e, f). Therefore, these results highlight the role of MTCH2 in glioma cell survival, implying that MTCH2 is a potential target for intervention.

## Discussion

**Fig. 7:**
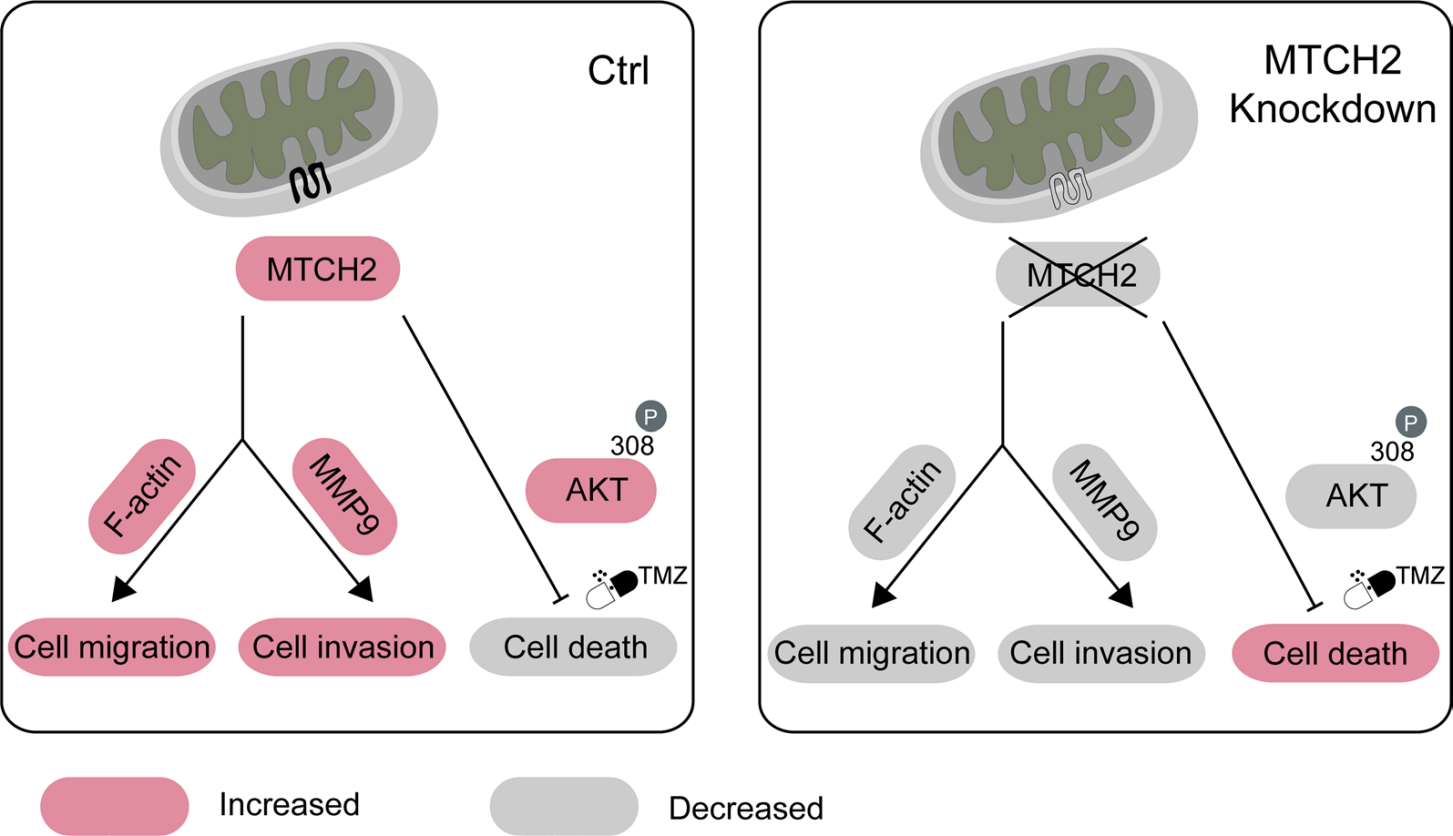
Model. A schematic demonstrates that MTCH2 regulates cell invasion/apoptosis of malignant gliomas. Silencing of MTCH2 expression impairs cell migration via F-actin depolymerization, decreases cell invasion via MMP-9 pathway, and represses pro-survival AKT signaling to promote TMZ-induced cell death

Mitochondrial dysfunction has long been appreciated as a molecular hallmark of malignant gliomas, and the underlying mechanisms have been popular targets for promising therapeutic interventions (Michelakis et al. 2010; Martinez-Outschoorn et al. 2017). The present study demonstrates that MTCH2, a critical protein in the mitochondrial outer membrane, regulates glioma malignancy including tumor migration and chemoresistance. Silencing of MTCH2 in glioma cells reduced cell migration/invasion and rendered cells more susceptible to apoptosis induced by temozolomide (Fig. 7).

Mitochondrion is surrounded by two membranes, and thus a large number of transport proteins are needed to link the biochemical pathways of the cytosol and mitochondrial matrix. This transport system is comprised of mitochondrial translocases of the outer/inner membrane (TOMs/TIMs), mitochondrial pyruvate carriers (MPCs), and mitochondrial carriers etc. (Lytovchenko and Kunji 2017). MTCH2 is a member of mitochondrial carrier family and regulates cell apoptosis by modulating the activity of mitochondrial permeability transition pore (Alcala et al. 2008). Previous studies examined the role of MTCH2 in several types of tumors, including mammary carcinomas (Arigoni et al. 2013; Leibowitz-Amit et al. 2006) and acute myeloid leukemia (AML) (Khan et al. 2020). Intriguingly, the role of MTCH2 in these tumors were opposite in breast cancer and AML. Our study demonstrates that MTCH2 is highly expressed in malignant gliomas. We noted that the mutation frequency of MTCH2 is low in glioma (less than 4% from cBioPortal, http://www.cbioportal.org/). Therefore, we propose that altered gene expression (not genomic mutation) of MTCH2 is the major way to regulate glioma malignancy.

Glioma has a strong glycolytic phenotype, and a number of the molecular abnormalities that occur in glioma are known to suppress mitochondrial OXPHOs and promote aerobic glycolysis (Strickland and Stoll 2017; Lokody 2014). Indeed, glycolytic enzymes have direct anti-apoptotic actions, and decreased mitochondrial function is associated with inhibition of mitochondria dependent apoptosis. MTCH2 is a repressor of mitochondrial metabolism in hematopoietic system, and loss of MTCH2 increases mitochondrial OXPHOs, accompanied with an increase in mitochondrial size, ATP production and consequently ROS accumulation (Maryanovich et al. 2015). Consistent with this notion, our findings expand current knowledge of MTCH2 in human gliomas. We showed that MTCH2 knockdown increases products of lipid peroxidation, 4-HNE, as well as the susceptibility to TMZ-induced cell death. Based on these results, we propose that MTCH2 knockdown may activate OXPHOs in glioma cells, and render cells more susceptible TMZ-induced apoptosis.

## Conclusion

In summary, our work identifies the role of MTCH2 in malignant gliomas. Glioma cells with reduced MTCH2 expression have lowered invasion property and are sensitive to temozolomide. Our results supplement current understanding in the mitochondrial biology of gliomas, and provide a potential target for future intervention.

## Supplementary Information

**Additional file 1: Table S1.** Patient information (TCGA and CGGA). **Table S2.** Patient information (our cohort).

**Additional file 2: Figure S1. **Kaplan-Meier survival analysis for human glioma datasets (TCGA and CGGA). **Figure S2**. Protein quantifications relative to Fig. 3.

## Data Availability

All relevant data are available from the corresponding authors, upon reasonable request.

## Abbreviations

MTCH2: Mitochondrial carrier homolog 2
GBM: Glioblastoma
TMZ: Temozolomide
OXPHOs: Oxidative phosphorylations
TCGA: The Cancer Genome Atlas Research Network
CGGA: The Chinese Glioma Genome Atlas
4-HNE: 4-Hydroxy-2-nonenal
ROS: Reactive oxygen species
