# Supplementary material for: Inhibition of mitochondrial carrier homolog 2 (MTCH2) suppresses tumor invasion and enhances sensitivity to temozolomide in malignant glioma
Source: Mol Med. 2021 Jan 28;27:7. doi: 10.1186/s10020-020-00261-4 (PMC7842075; doi:10.1186/s10020-020-00261-4)
Supplement: Supplementary file 2 — Additional file 2: Figure S1. Kaplan-Meier survival analysis for human glioma datasets (TCGA and CGGA). Figure S2. Protein quantifications relative to Fig. 3. [file 10020_2020_261_MOESM2_ESM.doc]

**Additional Material**

**Inhibition of Mitochondrial Carrier Homolog 2 (MTCH2) Suppresses Tumor Invasion and Enhances Sensitivity to Temozolomide in Malignant Glioma**

**Authors:** Qiuyun Yuan^1,#^, Wanchun Yang^1,#^, Shuxin Zhang^1^, Tengfei Li^1^, Mingrong Zuo^1^, Xingwang Zhou^1^, Junhong Li^1^, Mao Li^1^, Xiaoqiang Xia^1^, Mina Chen^1^,*, Yanhui Liu^1^,*

**Affiliation:**

^1^Department of Neurosurgery, State Key Laboratory of Biotherapy, West China Hospital, Sichuan University, Chengdu 610041, People’s Republic of China.

^#^These authors contributed equally to this work.

*Correspondence: Mina Chen (chenmina2010@scu.edu.cn) and Yanhui Liu ([liuyh@scu.edu.cn](mailto:liuyh@scu.edu.cn)), Department of Neurosurgery, West China Hospital, Sichuan University, 37 Guoxue Avenue, Chengdu, 610041, Sichuan Province, People’s Republic of China.

**This PDF file includes:**

Additional Figure 1-2

**Additional Figures and Legends**


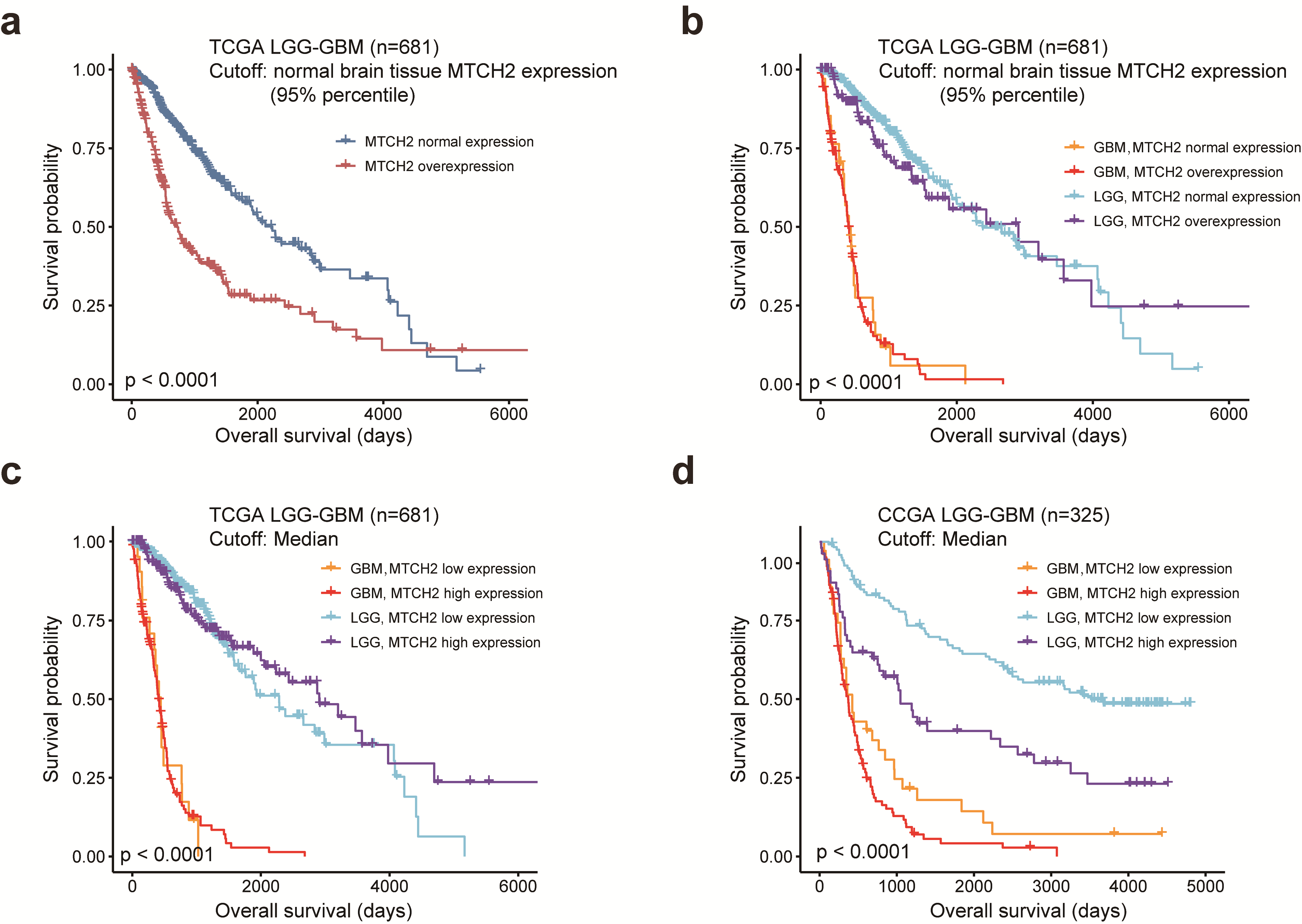
**Fig. S1 Kaplan-Meier survival analysis for human glioma datasets (TCGA and CGGA); relative to Fig. 1.**

**(a-d)** Survival analysis using clinical information from TCGA (a-c) and CGGA (d) dataset. Patients are divided into low and high MTCH2 groups by normal brain tissue MTCH2 expression (95% percentile) (a-b), or median expression level (c-d).


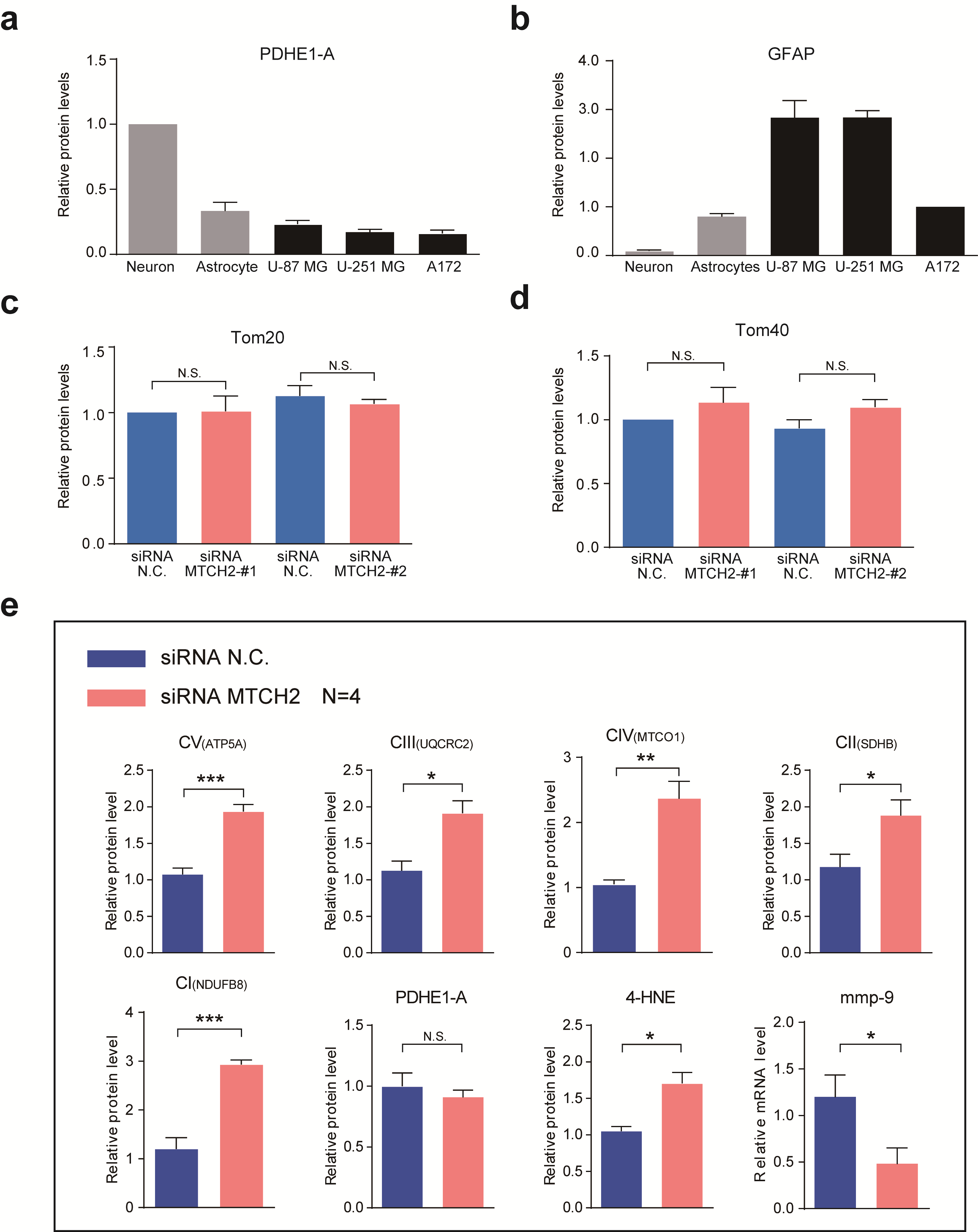
**Fig. S2 Protein quantifications relative to Fig. 3.**

**(a-b)** Quantifications showing the expression pattern of PDHE1-A and GFAP in glioma cells compared with primary neurons and astrocytes. **(c-d)** Quantifications showing that MTCH2 knockdown did not alter protein levels of Tom20 and Tom40 in in A172 cells. **(e)** Quantifications showing the increased mitochondrial respiratory proteins, 4-HNE levels, and decreased MMP-9 mRNA level by MTCH2 knockdown in A172 cells.
